# Changes in *Bemisia tabaci* feeding behaviors caused directly and indirectly by cucurbit chlorotic yellows virus

**DOI:** 10.1186/s12985-019-1215-8

**Published:** 2019-08-22

**Authors:** Shaohua Lu, Mingshun Chen, Jingjing Li, Yan Shi, Qinsheng Gu, Fengming Yan

**Affiliations:** 1 College of Plant Protection, Henan Agricultural University, Zhengzhou, 450002 Henan China; 2 School of Food Science and Technology, Henan University of Technology, Zhengzhou, 450001 Henan China; 3 Department of Entomology, Kansas State University, Manhattan, KS 66506 USA; 4 Chinese Academy of Agricultural Science, Zhengzhou Fruit Research Institute, Zhengzhou, 410100 China

**Keywords:** *Bemisia tabaci*, Cucurbit chlorotic yellows virus, Electrical penetration graph, Feeding behaviors, Semipersistent virus

## Abstract

**Background:**

Plant viruses can affect vector’s behaviors in order to enhance viral transmission. Cucurbit chlorotic yellows virus (CCYV) (genus *Crinivirus*) is an emergent RNA plant virus and is transmitted specifically by biotypes B and Q of tobacco whitefly, *Bemisia tabaci* (Gennadius), in a semipersistent manner.

**Methods:**

We used the electrical penetration graph (EPG) to investigate the effect of CCYV on the feeding behaviors of *B. tabaci* biotypes B and Q.

**Results:**

CCYV could affect, both directly and indirectly, the feeding behaviors of *B. tabaci* to various degrees, depending on biotypes and sexes of the insect. CCYV showed stronger direct effects on biotype Q than on biotype B in terms of increased non-phloem probing and phloem salivation. CCYV increased non-phloem probing and phloem salivation more on females than on males of biotype Q, and increased phloem salivation more on females than on males of biotype B. CCYV had stronger indirect effects, via virus-infested plants, on biotype B than on biotype Q by enhancing phloem sap ingestion and feeding bouts. CCYV increased non-phloem probing and feeding bouts more on males than on females of biotype B, and decreased phloem sap ingestion more on males than on females on biotype Q indirectly.

**Conclusions:**

The results clearly indicated that CCYV affects the feeding behaviors of *B. tabaci*, which may lead to increased ability of the *B. tabaci* for CCYV transmission.

## Background

The feeding behaviors of vector insects plays a critical role in transmitting plant viruses from one host to another over distantly-located regions [1–4]. Plant viruses can manipulate vector insects by directly influencing the behavior and physiology of the insects [5]. For example, western flower thrips, *Frankliniella occidentalis*, carrying tomato spotted wilt virus (TSWV) make more non-ingestive probes to transmit the virus into plant cells. Tobacco whitefly *Bemisia tabaci* with tomato yellow leaf curl virus (TYLCV) spend more time in phloem salivating and ingesting sap, resulting in enhanced viral acquisition and transmission [6, 7]. Tomato yellow leaf curl China virus (TYLCCNV) affects *B. tabaci* behaviors relevant to effective and rapid sap ingestion on virus-infected tobacco plant [8]. Plant viruses can also affect behaviors of vector insects indirectly, for example, by altering host plant characteristics such as color, nutrition and volatiles [2, 9–11]. Different biotypes of *B. tabaci* are attracted to TYLCV-infected tomato plants to a greater degree [12], and conduct probing more quickly with a greater number of phloem feeding bouts on TYLCV-infected plants [13]. Moreover, some studies indicate that the growth and development of *B. tabaci* on cassava mosaic geminiviruses (CMG)-infected plants have more eggs laid than on CMG-free plants [14]. After cultured for 56 days, the population density of *B. tabaci* biotype B on tobacco curly shoot virus (TbCSV) and TYLCCNV-infected plants had 2 times and 13 times higher than those on healthy plants, respectively [15]. However, some studies show the opposite results that *B. tabaci* males and females had shorter longevity on cotton leaf curl virus (CLCuV) infected plants than on healthy plants [16]. These are just some examples indicating that the impact of interactions among viruses, insect vectors, and host plants on viral pandemics has attracted more and more attention in recent years [17, 18].

*Bemisia tabaci* (Gennadius) (Hemiptera: Aleyrodidae) is considered as a cryptic species with at least 39 morphologically indistinguishable biotypes, which are often reproductively isolated [19–21]. Biotype B (also referred to as Middle East-Asia Minor 1) and biotype Q (also referred to as Mediterranean) are the two most invasive and destructive in *B. tabaci* [19]. In the past 30 years, *B. tabaci* biotypes B and Q have invaded many countries worldwide and displaced some indigenous cryptic biotypes [19]. Both biotypes B and Q can seriously damage plants by feeding upon phloem sap and secreting honeydew, which can result in fungal growth on damaged plant tissues. In addition, *B. tabaci* can transmit plant viruses, some of which could be devastating to crop plants [22, 23]. To date, more than 200 plant virus species have been reported to be transmitted by *B. tabaci* [24–26]. Viruses in the genera of *Begomovirus*, *Crinivirus*, *Ipomovirus*, *Carlavirus* and *Torradovirus* can be transmitted by *B. tabaci*. Viral epidemic outbreak of whitefly-transmitted viruses in various regions is often a result of high population densities, especially high abundance of biotypes B and Q [22, 27, 28].

Cucurbit chlorotic yellows virus (CCYV) (genus *Crinivirus*) is a single-stranded, positive-sense plant RNA virus, composed of RNA1 and RNA2, and is transmitted by *B. tabaci* biotypes B and Q in a semipersistent manner [29]. CCYV can infect a wide range of plants, including melon, cucumber, watermelon, loofah plants, pumpkin, *Nicotiana benthamiana* and other plant species. CCYV causes symptoms on infested plants from chlorotic leaf spots to completely yellowish leaves [29, 30], resulting in serious yield losses. CCYV was first described in Japan in 2004, and since then the virus has also been found in Taiwan [30], China mainland [31, 32], Sudan [33], Lebanon [34], Iran [35], Greece [36], Saudi Arabia [37] and California [38]. To date, few studies are available on the interactions of semipersistent viruses, *B. tabaci*, and plants. Direct effects of CCYV on feeding behaviors of its vector *B. tabaci* biotypes B and Q have been observed on cotton plants (host for *B. tabaci*, but not CCYV) [39]. Here we report that CCYV can influence feeding behaviors of its vector insect directly, or indirectly via the influence of CCYV-infected cucumber plants. Cucumber is a host plant for both *B. tabaci* and CCYV.

Electrical penetration graph (EPG) is a reliable tool to study the feeding behaviors of piercing-sucking insects [40, 41]. EPG waveforms can reveal details of probing behaviors of insects such as stylet tip positions inside plant tissues (epidermis, mesophyll, phloem, or xylem) and relevant insect activities (intercellular probing, short intracellular sap sampling, sheath salivation, watery salivation and sap ingestion in phloem) [42]. This type of data can help to gain information on piercing sucking insects and plant interactions [43], plant resistant mechanisms [44], location of potential antifeedants or feeding stimulants in plant tissues [45], and transmission processes of the plant viruses and other pathogens by vector insects [4, 39, 46].

In this study, we used EPG to compare feeding behaviors of non-viruliferous and viruliferous *B. tabaci* biotypes B and Q on non-viruliferous and viruliferous cucumber plants. We found that CCYV could directly impact the feeding behaviors of *B. tabaci*, such as probing and salivation, in a manner consistent with accelerated viral spread. CCYV could also influence feeding behaviors of *B. tabaci* indirectly by causing changes in host plants, which then impact *B. tabaci*. These results indicated that CCYV could affect, both directly and indirectly, the feeding behavior of *B. tabaci* to various degrees, depending on biotypes and sexes of the vector insect.

## Materials and methods

### Plants

Cucumber (*Cucumis sativus* L. cv. Bojie-107) plants were grown in pots (d = 12.5 cm) in a greenhouse under a photoperiod 16:8 LD, temperature 26 ± 1 °C, and relative humidity 70 ± 0.5%. To obtain viruliferous cucumber plants, *Agrobacterium tumefaciens*-mediated CCYV clones were used to inoculate cucumber plants at one true-leaf stage [47]. About 25 days later, the infection status of cucumber plants was determined based on the symptom of yellowing and chlorotic leaf spots. Infection was further confirmed by reverse transcription-polymerase chain reaction [39]. All plants were maintained in separate insect-proof cages (60 cm × 40 cm × 80 cm) in greenhouse under the same conditions. Cucumber plants at 4 true-leaf stage were used for all experiments.

### *B. tabaci* populations

*B. tabaci* biotypes B and Q were maintained on non-viruliferous cucumber (*Cucumis sativus* L. cv. Bojie-107) plants for many years in insect-proof cages under conditions as above. The purity of biotypes B and Q populations was monitored every 1–2 generations by using the biomarkers of the *mitochondrial cytochrome oxidase I* (*mtCOI*) genes [48, 49].

Non-viruliferous and viruliferous *B. tabaci* colonies were established by transferring about 300 pairs of adult males and females of biotypes B and Q from the laboratory populations into insect-proof cages each with two virus-free or CCYV-infected cucumber plants, respectively. Non-viruliferous and viruliferous *B. tabaci* colonies were maintained for 2 generations in a greenhouse under the conditions described previously. Starting from the third generation, we randomly selected newly emerged *B. tabaci* male and female adults from each colony for use in the experiments.

### EPG recording

A 4-channel direct-current EPG system (Wageningen University, the Netherlands) was used to monitor the feeding behaviors of *B. tabaci*. Prior to a recording, a gold wire (1.5 cm long and 12.5 μm in diameter) was attached to the pronotum of an insect using a drop of water-based silver glue. Each wired insect was starved for ca. 20 min before connected to the Giga-4 probe input and placed onto the abaxial surface of the third leaf of cucumber plant. Six hours of EPGs were continuously recorded for each replicate, which was defined as one adult *B. tabaci* feeding on one plant. All the recoding experiments were finished in an electrically grounded Faraday cage to block electric fields. All experiments were carried out in a quiet room under temperature 26 ± 1 °C, relative humidity 70 ± 0.5%, and 1000 lx artificial light. EPG signals were digitized with a DI-710-UL analogue-to-digital converter (Dataq Instruments, Akron, OH, the USA), and the output was acquired and stored with Stylet+ (d / a) for Windows software (Wageningen University, the Netherlands), and data were analyzed with this software after data conversion.

EPG waveforms were categorized as previously described [7, 50]. Four distinct waveforms were identified in this study: pathway [C, showing insect stylet activities from epidermis to the phloem, including intercellular penetration and sheath salivation, as well as, if occur, penetration difficulties (F waveform) and xylem sap ingestion (G waveform)]; potential drop (intracellular puncture) [pd], and the phloem phase salivation into a sieve element [E1] and ingestion of sieve element sap [E2]. The time from the start to the end of each waveform was recorded and exported by using Stylet+ software. Based on the information described above, we selected 6 non-phloem phase variables and 8 phloem phase variables for analysis and comparison of *B. tabaci* feeding behaviors of (1) non-viruliferous biotypes B and Q feeding on non-viruliferous cucumber plants, (2) viruliferous biotypes B and Q feeding on non-viruliferous cucumber plants, and (3) viruliferous biotypes B and Q feeding on viruliferous cucumber plants.

### Data analysis

SPSS Statistics 20.0 (IBM Corp., Armonk, NY) was used in all statistical analyses. Significant differences were tested at the 0.05 or 0.01 level. Data were log10-transformed when it did not fit a normal distribution after checked normality and homogeneity of variance. Independent-Samples *t*-test was conducted to compare the means of data obtained with biotypes B and Q, separately, in each treatment, including combined data and data after separation into male’s and female’s. One-way ANOVA was used to analyze measurements of the feeding behaviors of biotypes B or Q from the three treatments, including combined data and data after separation into male’s and female’s. Multivariate analysis of variance was carried out to analyze potential interactions among biotypes of vector insects, sexes (male and female) of each biotype, insect status (non-viruliferous and viruliferous insects), and plant status (non-viruliferous and viruliferous plants). Means were compared by least significant difference (Tukey’s) tests.

## Results

We conducted EPG analyses on non-viruliferous *B. tabaci* feeding on non-viruliferous cucumber plants, viruliferous *B. tabaci* feeding on non-viruliferous cucumber plants, and viruliferous *B. tabaci* feeding on viruliferous cucumber plants. A total of 231 successful EPG recordings were obtained, including 82 for non-viruliferous *B. tabaci* on non-viruliferous cucumber plants (22 replicates for biotype B males, 22 replicates for biotype B females, 19 replicates for biotype Q males and 19 replicates for biotype Q females), 72 for viruliferous *B. tabaci* on non-viruliferous cucumber plants (18 replicates for biotype B males, 18 replicates for biotype B females, 18 replicates for biotype Q males and 18 replicates for biotype Q females), and 77 for viruliferous *B. tabaci* on viruliferous cucumber plants (18 replicates for biotype B males, 22 replicates for biotype B females, 18 replicates for biotype Q males and 19 replicates for biotype Q females).

### Overall direct effects of CCYV on feeding behaviors of biotypes B and Q

The direct effects of CCYV on feeding behaviors of *B. tabaci* biotypes B and Q were obtained by comparing data obtained with viruliferous *B. tabaci* (data from viruliferous *B. tabaci* on non-viruliferous plants) with data under control conditions (non-viruliferous *B. tabaci* on non-viruliferous plants).

#### Non-phloem feeding behaviors

**Fig. 1:**
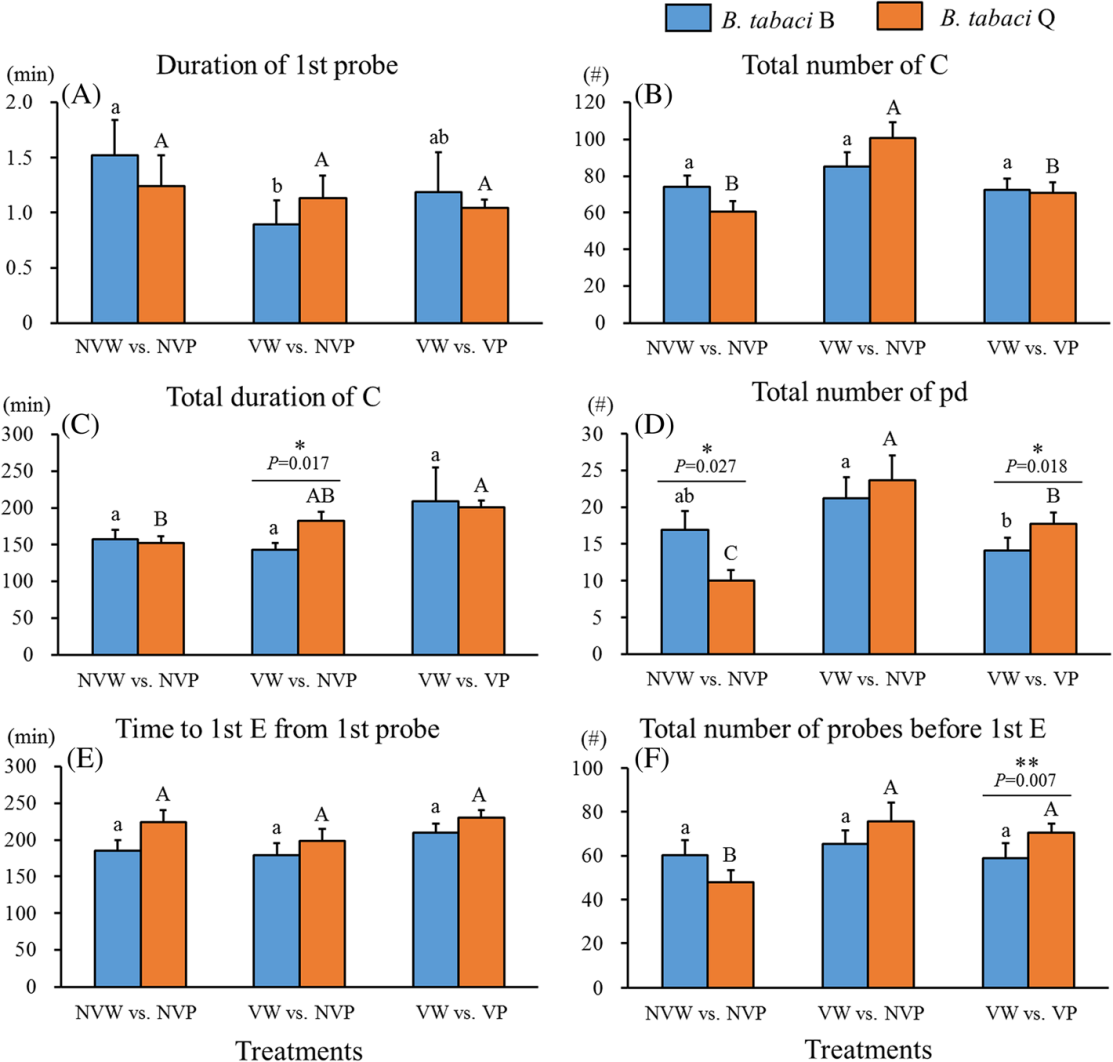
Effects of CCYV on non-phloem EPG variables of *Bemisia tabaci* biotypes B and Q. The three treatments include: non-viruliferous *B. tabaci* whitefly feeding on non-viruliferous cucumber plants (NVW vs. NVP), viruliferous *B. tabaci* whitefly feeding on non-viruliferous cucumber plants (VW vs. NVP), and viruliferous *B. tabaci* whitefly feeding on viruliferous cucumber plants (VW vs. VP). Data are presented as means ± SE. Asterisks * or ** indicate statistically significant differences between biotypes B and Q on plants under the same treatments at *P* < 0.05 or *P* < 0.01. Lowercase and uppercase letters represent the comparison of biotype B (a, b, c) or biotype Q (A, B, C) on plants under different treatments. Letters above the bars indicate statistically significant among treatments (Tukey test, *P* < 0.05). EPG waveforms: C = pathway; pd. = potential drop (intracellular puncture); E1 = phloem salivary secretion; E2 = phloem sap ingestion. E = E1 + E2

The direct impact of CCYV on *B. tabaci* biotypes B and Q was different. Specifically, CCYV shortened first probe of biotype B (Fig. 1a). However, CCYV resulted in 1.7 times more total number of pathway (Fig. 1b), 2.4 times more total number of potential drop (intracellular puncture) (Fig. 1d), and 1.6 times more total number of probes before phloem of biotype Q (Fig. 1f). No significant difference was observed in feeding behaviors between non-viruliferous biotypes B and Q in non-phloem phase except biotype B had higher total number of potential drop (intracellular puncture) (Fig. 1d) than biotype Q. The difference in potential drop between these two biotypes disappeared after CCYV carrying because of the increase in potential drop associated with biotype Q after CCYV carrying (Fig. 1d). The differential impact of CCYV between these two biotypes also resulted in significantly longer total duration of pathway (Fig. 1c) with biotype Q than biotype B. No significant changes were found on the variable of time to phloem from 1 st probe (Fig. 1e) between non-viruliferous and viruliferous biotypes B and Q on non-viruliferous cucumber plants.

#### Phloem feeding behaviors

**Fig. 2:**
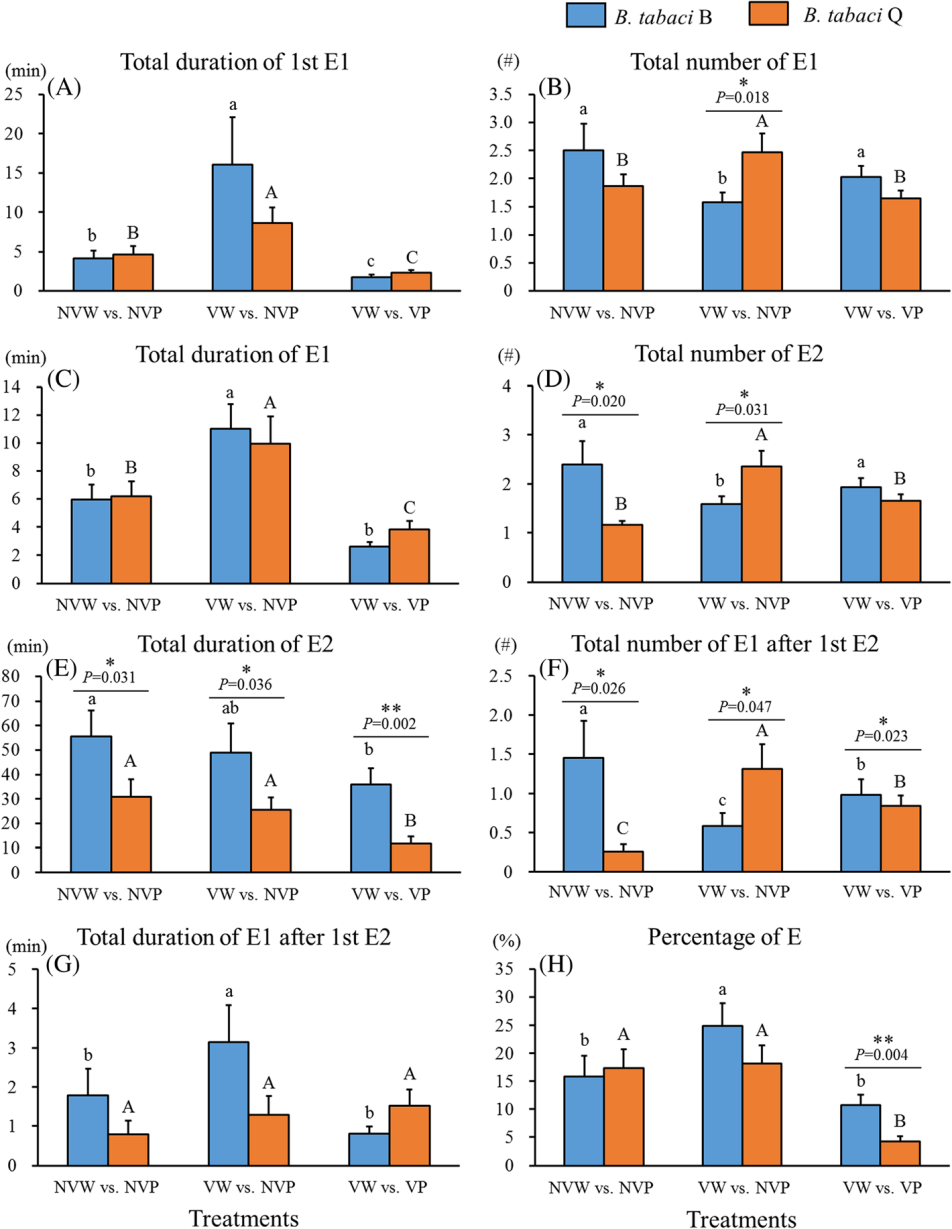
Effects of CCYV on phloem EPG variables of *Bemisia tabaci* biotypes B and Q. The three treatments include: non-viruliferous *B. tabaci* whitefly feeding on non-viruliferous cucumber plants (NVW vs. NVP), viruliferous *B. tabaci* whitefly feeding on non-viruliferous cucumber plants (VW vs. NVP), and viruliferous *B. tabaci* whitefly feeding on viruliferous cucumber plants (VW vs. VP). Data are presented as means ± SE. Asterisks * or ** indicate statistically significant differences between biotypes B and Q on plants under the same treatments at *P* < 0.05 or *P* < 0.01. Lowercase and uppercase letters represent the comparison of biotype B (a, b, c) or biotype Q (A, B, C) on plants under different treatments. Letters above the bars indicate statistically significant among treatments (Tukey test, *P* < 0.05) Percentage of E (%) = equals the percentage of total duration of E (E1 + E2). EPG waveforms: E1 = phloem salivary secretion; E2 = phloem sap ingestion. E = E1 + E2

Overall, biotype Q was affected by CCYV to a greater degree than biotype B. The common effect of CCYV on both biotypes B and Q included ~ 2 times longer total duration of 1st salivation (Fig. 2a) and ~ 1.7 times longer total duration of salivation (Fig. 2c). The specific effects of CCYV on biotype B included reduced total number of salivation (37%, Fig. 2b), reduced total number of sap ingestion (34%, Fig. 2d) and reduced total number of salivation after 1st sap ingestion (60%, Fig. 2f); but increased total duration of salivation after 1st sap ingestion (1.8 times, Fig. 2g), and increased percentage of phloem phase (salivation + ingestion) (1.6 times, Fig. 2h). The specific effects of CCYV on biotype Q included increased total number of salivation (1.3 times, Fig. 2b), increased total number of sap ingestion (2 times, Fig. 2d), and increased total number of salivation after 1st sap ingestion (5 times, Fig. 2f). Non-viruliferous biotype Q had only 50% of total number of sap ingestion (Fig. 2d) and only 18% of total number of salivation after 1st sap ingestion (Fig. 2f) in comparison with the corresponding variables of non-viruliferous biotype B. CCYV increased significantly the total number of E1 (1.6 times, Fig. 2b), total number of sap ingestion (1.5 times, Fig. 2d), total number of salivation after 1st sap ingestion (2.3 times, Fig. 2f) of biotype Q than biotype B. However, because biotype Q had much lower total duration of sap ingestion (Fig. 2e) than biotype B without CCYV carrying, it still had only 52% of total duration of sap ingestion (Fig. 2e) of biotype B even after CCYV carrying.

### Direct impact of CCYV on feeding behaviors of biotype B males and females

#### Non-phloem feeding behaviors

CCYV didn’t cause significant change in non-phloem feeding behaviors of biotype B males on non-viruliferous plants. However, CCYV caused a significant change in feeding behaviors of biotype B females. Specifically, CCYV reduced the duration of 1st probe (51%, Table 1, Variable 1) of biotype B females. No significant difference was observed in feeding behaviors between non-viruliferous biotype B males and females in non-phloem phase. Because of the differential impact of CCYV on biotype B males and females, females exhibited 1.7 times more total number of pathway (Table 1, Variable 2), 1.5 times longer time to phloem from 1st probe (Table 1, Variable 5) and 1.7 times more total number of probes before 1st phloem phase (Table 1, Variable 6) than males.

**Table 1:** EPG variables of different sexes of different treatments of *Bemisia tabaci* biotype B

Variables	Sex	NVW vs. NVP^1^	VW vs. NVP	VW vs. VP
Non-phloem variables				
1. Duration of 1st probe (min)	♂	1.23 ± 0.40^2^a	0.90 ± 0.21a^3^	0.62 ± 0.08b
	♀	1.81 ± 0.50a	0.89 ± 0.39b	1.65 ± 0.64ab*^4^
2. Total number of C (#)	♂	71.64 ± 10.03a	63.61 ± 6.03a	58.28 ± 7.59a
	♀	76.32 ± 7.87a	106.28 ± 12.74a**	83.82 ± 9.04a*
3. Total duration of C (min)	♂	150.99 ± 15.17b	138.37 ± 13.80b	245.68 ± 10.26a*
	♀	163.73 ± 20.13a	147.68 ± 13.61a	179.10 ± 12.39a
4. Total number of pd. (#)	♂	18.41 ± 4.00ab	22.33 ± 4.50a	12.61 ± 2.89b
	♀	15.41 ± 3.18a	20.06 ± 3.47a	15.27 ± 2.28a
5. Time to 1st E from 1st probe (min)	♂	178.39 ± 18.68a	145.55 ± 23.20a	176.39 ± 19.40a
	♀	191.29 ± 23.45a	213.20 ± 18.06a*	236.93 ± 14.81a*
6. Total number of probes before 1st E (#)	♂	57.91 ± 9.57a	47.94 ± 6.19a	38.56 ± 6.47a
	♀	62.68 ± 9.42a	82.67 ± 9.49a**	75.41 ± 10.39a*
Phloem variables				
7. Total duration of 1st E1 (min)	♂	4.21 ± 1.36b	12.98 ± 3.03a	1.59 ± 0.38c
	♀	4.14 ± 1.32b	19.10 ± 11.79a	1.93 ± 0.38c
8. Total number of E1 (#)	♂	1.82 ± 0.29b	1.11 ± 0.08b	2.67 ± 0.32a**
	♀	3.18 ± 0.91a	2.06 ± 0.29a**	1.50 ± 0.16a
9. Total duration of E1 (min)	♂	6.16 ± 1.79b	13.59 ± 2.91a**	2.73 ± 0.57b
	♀	5.80 ± 1.20ab	8.45 ± 1.83a	2.57 ± 0.34b
10.Total number of E2 (#)	♂	1.68 ± 0.25b	1.21 ± 0.08b	2.56 ± 0.32a**
	♀	3.09 ± 0.90a	2.36 ± 0.19a*	1.41 ± 0.13b
11. Total duration of E2 (min)	♂	46.83 ± 12.60ab	70.80 ± 21.69a*	33.06 ± 11.93b
	♀	64.52 ± 16.79a	27.28 ± 7.21b	38.14 ± 7.83b
12. Total number of E1 after 1st E2 (#)	♂	0.73 ± 0.26b	0.11 ± 0.08c	1.61 ± 0.35a**
	♀	2.18 ± 0.91a	1.06 ± 0.29a**	0.45 ± 0.16a
13. Total duration of E1 after 1st E2 (min)	♂	1.92 ± 1.30a	0.61 ± 0.42a	1.13 ± 0.30a*
	♀	1.65 ± 0.50b	5.67 ± 1.67a**	0.54 ± 0.19b
14. Percentage of E (%)^5^	♂	0.24 ± 0.06c	31.12 ± 6.85a*	9.94 ± 3.28b
	♀	31.42 ± 5.67a**	18.76 ± 3.85ab	11.31 ± 2.18b

^1^The three treatments include: non-viruliferous *B. tabaci* whitefly feeding on non-viruliferous cucumber plants (NVW vs. NVP), viruliferous *B. tabaci* whitefly feeding on non-viruliferous cucumber plants (VW vs. NVP), and viruliferous *B. tabaci* whitefly feeding on viruliferous cucumber plants (VW vs. VP). ^2^Data are means ± SE. ^3^Letters immediately after the mean values represent the comparison of biotype B on plants under different treatments. Letters after the mean values indicate statistically significant among treatments (Tukey test, *P* < 0.05). ^4^Asterisks * or ** indicate a statistically significant difference between biotype B males and females on plants under the same treatments at *P* < 0.05 or *P* < 0.01. ^5^Percentage of E (%) = equals the percentage of total duration of E (E1+E2). EPG waveforms: C = pathway; pd = potential drop (intracellular puncture); E1 = phloem salivary secretion; E2 = phloem sap ingestion. E=E1 + E2

#### Phloem feeding behaviors

CCYV caused significant changes in phloem feeding behaviors of both *B. tabaci* biotype B males and females. Overall CCYV prolonged total duration of 1st salivation (Table 1, Variable 7) of both males and females, but resulted in longer total duration of salivation (Table 1, Variable 9) and higher percentage of phloem phase (salivation + ingestion) (Table 1, Variable 14) of biotype B males, even though biotype B males had only 15% of total number of salivation after 1st sap ingestion (Table 1, Variable 12) in comparison with non-viruliferous males. The specific effects of CCYV on biotype B females included, reduced total duration of sap ingestion (58%, Table 1, Variable 11) and increased total duration of salivation after 1st sap ingestion (3.4 times, Table 1, Variable 13). No significant difference was observed in feeding behaviors between non-viruliferous biotypes B males and females in phloem phase except biotype B females had higher percentage of phloem phase (salivation + ingestion) (Table 1, Variable 14) than biotype B males. Because of the differential impact of CCYV infection on biotypes B males and females, CCYV increased total number of salivation (1.9 times, Table 1, Variable 8), total number of sap ingestion (2 times, Table 1, Variable 10), total number of salivation after 1st sap ingestion (9.6 times, Table 1, Variable 12) and total duration of salivation after 1st sap ingestion (9.3 times, Table 1, Variable 13) of biotype B females, even though biotype B females exhibited only 62% of total duration of salivation (Table 1, Variable 9), 39% of total duration of sap ingestion (Table 1, Variable 11) and 60% of percentage of phloem phase (salivation + ingestion) (Table 1, Variable 14) compared with those parameters obtained with biotype B males.

### Direct impact of CCYV on feeding behaviors of biotype Q males and females

#### Non-phloem feeding behaviors

CCYV caused significant changes in non-phloem probing of both *B. tabaci* biotype Q males and females. The common effect of CCYV on biotype Q males and females included ~ 1.7 times more total number of pathway (Table 2, Variable 2) and 2.2 times more total number of potential drop (intracellular puncture) (Table 2, Variable 4). The specific effects of CCYV on biotype Q males included increased total duration of pathway (1.4 times, Table 2, Variable 3) and total number of probes before phloem (1.7 times, Table 2, Variable 6). No significant difference was observed in feeding behaviors between non-viruliferous biotype Q males and females in non-phloem phase except biotype Q females had 1.4 times longer total duration of pathway (Table 2, Variable 3) than biotype Q males. Non-phloem phase feeding behaviors did not significantly differ between biotypes Q males and females after CCYV carrying.

**Table 2:** EPG variables of different sexes of different treatments of *Bemisia tabaci* biotype Q

Variables	Sex	NVW vs. NVP ^1^	VW vs. NVP	VW vs. VP
Non-phloem variables				
1. Duration of 1st probe (min)	♂	1.41 ± 0.51^2^a	1.16 ± 0.39a^3^	1.23 ± 0.08a*^4^
	♀	1.08 ± 0.21a	1.10 ± 0.20a	0.86 ± 0.13a
2. Total number of C (#)	♂	61.63 ± 8.67b	101.33 ± 12.48a	68.39 ± 10.18ab
	♀	59.79 ± 7.21b	100.44 ± 11.73a	72.89 ± 6.33ab*
3. Total duration of C (min)	♂	126.60 ± 11.80b	178.56 ± 21.90a	213.15 ± 13.21a
	♀	177.65 ± 13.06a*	184.63 ± 13.07a	189.43 ± 15.65a
4. Total number of pd. (#)	♂	9.74 ± 1.88b	21.56 ± 4.28a	17.67 ± 1.31ab
	♀	10.21 ± 2.44b	25.67 ± 5.44a	17.74 ± 2.78ab
5. Time to 1st E from 1st probe (min)	♂	224.56 ± 22.28ab	188.72 ± 20.42b	246.95 ± 6.50a
	♀	223.36 ± 24.63a	207.82 ± 25.64a	213.85 ± 19.11a
6. Total number of probes before 1st E (#)	♂	46.84 ± 6.49b	80.33 ± 10.66a	83.89 ± 3.94a**
	♀	48.74 ± 9.04a	70.78 ± 13.91a	58.11 ± 5.03a
Phloem variables				
7. Total duration of 1st E1 (min)	♂	3.40 ± 1.34b	6.35 ± 1.49a	2.71 ± 0.39b
	♀	5.85 ± 1.70b	10.87 ± 3.70a	1.99 ± 0.50c
8. Total number of E1 (#)	♂	1.89 ± 0.26a	2.39 ± 0.49a	1.56 ± 0.12a
	♀	1.84 ± 0.34a	2.56 ± 0.44a	1.74 ± 0.25a
9. Total duration of E1 (min)	♂	5.35 ± 1.45ab	7.56 ± 1.41a	4.49 ± 0.70b
	♀	7.05 ± 1.61b	12.39 ± 3.63a	3.23 ± 0.93c
10.Total number of E2 (#)	♂	1.32 ± 0.11a	2.17 ± 0.44a	1.56 ± 0.12a
	♀	1.00 ± 0.11b	2.56 ± 0.44a	1.74 ± 0.25ab
11. Total duration of E2 (min)	♂	30.19 ± 12.04a	27.59 ± 7.02a	7.19 ± 0.71b
	♀	31.25 ± 8.85a	23.49 ± 7.32a	15.88 ± 5.90a*
12. Total number of E1 after 1st E2 (#)	♂	0.42 ± 0.16a*	1.06 ± 0.45a	1.00 ± 0.12a
	♀	0.11 ± 0.07b	1.56 ± 0.44a	0.68 ± 0.25ab
13. Total duration of E1 after 1st E2 (min)	♂	0.99 ± 0.54a	1.04 ± 0.47a	1.88 ± 0.33a
	♀	0.56 ± 0.45a	1.52 ± 0.84a	1.20 ± 0.75a
14. Percentage of E (%)^5^	♂	17.25 ± 5.17a	21.85 ± 5.32a	3.06 ± 0.42a
	♀	17.51 ± 4.44a	14.43 ± 3.98a	5.31 ± 1.85b

^1^The three treatments include: non-viruliferous *B. tabaci* whitefly feeding on non-viruliferous cucumber plants (NVW vs. NVP), viruliferous *B. tabaci* whitefly feeding on non-viruliferous cucumber plants (VW vs. NVP), and viruliferous *B. tabaci* whitefly feeding on viruliferous cucumber plants (VW vs. VP). ^2^Data are means ± SE. ^3^Letters immediately after the mean values represent the comparison of biotype Q on plants under different treatments. Letters after the mean values indicate statistically significant among treatments (Tukey test, *P* < 0.05). ^4^Asterisks * or ** indicate a statistically significant difference between biotype Q males and females on plants under the same treatments at *P* < 0.05 or *P* < 0.01. ^5^Percentage of E (%) = equals the percentage of total duration of E (E1+E2). EPG waveforms: C = pathway; pd = potential drop (intracellular puncture); E1 = phloem salivary secretion; E2 = phloem sap ingestion. E=E1 + E2

#### Phloem feeding behaviors

CCYV exerted direct impact on phloem feeding behaviors of both males and females of *B. tabaci* biotype Q. CCYV prolonged total duration of 1st salivation (2 times, Table 2, Variable 7) of both biotype Q males and females. In addition, CCYV infection increased the total duration of salivation (1.8 times, Table 2, Variable 9), total number of sap ingestion (2.6 times, Table 2, Variable 10) and total number of salivation after 1st sap ingestion (14 times, Table 2, Variable 12) of biotype Q females. No significant difference was observed in feeding behaviors between non-viruliferous biotypes Q males and females in phloem phase except biotype Q females had only 26% of total number of salivation after 1st sap ingestion (Table 2, Variable 12) than biotype Q males. Because of the impact of CCYV on biotype Q males and females, the phloem phase feeding behaviors did not significantly differ between biotype Q males and females.

### Overall indirect effects of CCYV on feeding behaviors of biotypes B and Q

The indirect effects of CCYV on feeding behaviors of *B. tabaci* biotypes B and Q were defined as the effect of CCYV on vector insects via viruliferous plants. Specifically, indirect effects were obtained by comparing data from viruliferous *B. tabaci* feeding on viruliferous cucumber plants (VW vs. VP) with data obtained with viruliferous *B. tabaci* feeding on non-viruliferous cucumber plants (VW vs. NVP).

#### Non-phloem feeding behaviors

Viruliferous plants reduced the probing behaviors of both *B. tabaci* biotypes. Viruliferous plants shortened total number of potential drop (intracellular puncture) (40%, Fig. 1d) of both biotypes, and specifically reduced total number of pathway (30%, Fig. 1b) of biotype Q, whereas no significant impact was observed on biotype B. Because of the differential impact of viruliferous plants on biotypes B and Q, biotype Q exhibited 1.3 times more total number of potential drop (Fig. 1d) and 1.2 times more total number of probes before phloem (Fig. 1f) than biotype B on viruliferous plants. However, the time to phloem from 1st probe (Fig. 1e) did not significantly differ between viruliferous biotypes B and Q on non-viruliferous and viruliferous cucumber plants.

#### Phloem feeding behaviors

Viruliferous plants reduced the salivation in sieve tube elements of both *B. tabaci* biotypes. Overall viruliferous plants shortened total duration of 1st salivation (Fig. 2a), shortened total duration of salivation (Fig. 2c), and reduced percentage of phloem phase (salivation + ingestion) (Fig. 2h) of both biotypes B and Q. The specific effects of viruliferous plants on biotype B included: increased total number of salivation (1.3 times, Fig. 2b), increased total number of sap ingestion (1.2 times, Fig. 2d), increased total number of salivation after 1st sap ingestion (1.7 times, Fig. 2f); but reduced total duration of salivation after 1st sap ingestion (25%, Fig. 2g). The specific effects of viruliferous plants on biotype Q included: reduced total number of salivation (33%, Fig. 2b), reduced total number of sap ingestion (30%, Fig. 2d), reduced total number of salivation after 1st sap ingestion (64%, Fig. 2f), and reduced of total duration of sap ingestion (36%, Fig. 2e). Because of the differential impact of viruliferous plants on biotypes B and Q, biotype B had 3.1 times longer total duration of sap ingestion (Fig. 2e), 1.2 times more total number of salivation after 1st sap ingestion (Fig. 2f) and 2.5 times higher percentage of phloem phase (salivation + ingestion) (Fig. 2h) than biotype Q.

### Indirect impact of CCYV on feeding behaviors of biotype B males and females

#### Non-phloem feeding behaviors

Indirect impacts of CCYV on non-phloem feeding behaviors of *B. tabaci* biotype B males and females were different. Viruliferous plants reduced duration of 1st probe (33%, Table 1, Variable 1), reduced total number of potential drop (intracellular puncture) (44%, Table 1, Variable 4); but increased total duration of pathway (1.8 times, Table 1, Variable 3) of biotype B males. No significant indirect impact of CCYV on females was observed. Because of the differential impact of viruliferous plants on biotype B males and females, females had 2.7 times more duration of 1st probe (Table 1, Variable 1), 1.4 times more total number of total number of pathway (Table 1, Variable 2), 1.3 times more time to phloem from 1st probe (Table 1, Variable 5), 2 times total number of probes before 1st phloem phase (Table 1, Variable 6), but only 73% of total duration of pathway (Table 1, Variable 3) compared with those parameters obtained with males.

#### Phloem feeding behaviors

Overall viruliferous plants shortened total duration of 1st salivation (Table 1, Variable 7) and total duration of salivation (Table 1, Variable 9) of both *B. tabaci* biotype B males and females. The specific effects of viruliferous plants on biotype B males included: increased total number of salivation (2.4 times, Table 1, Variable 8), more total number of sap ingestion (2.1 times, Table 1, Variable 10) and more total number of salivation after 1st sap ingestion (14.5 times, Table 1, Variable 12); but reduced total duration of sap ingestion (53%, Table 1, Variable 11) and lowered percentage of phloem phase (salivation + ingestion) (68%, Table 1, Variable 14). The specific effects of viruliferous plants on biotype B females included: reduced total number of sap ingestion (68%, Table 1, Variable 10) and shortened total duration of salivation after 1st sap ingestion (90%, Table 1, Variable 13). Because of the differential impact of viruliferous plants on biotype B males and females, males exhibited 1.8 times more total number of salivation (Table 1, Variable 8), 1.8 times more total number of sap ingestion (Table 1, Variable 10), 3.6 times more total number of salivation after 1st sap ingestion (Table 1, Variable 12) and 2.1 times more total duration of salivation after 1st sap ingestion (Table 1, Variable 13) compared with the corresponding parameters obtained with females.

### Indirect impact of CCYV on feeding behaviors of biotype Q males and females

#### Non-phloem feeding behaviors

Viruliferous plants caused no statistically significant impact on *B. tabaci* biotype Q females, and only exhibited limited impact on biotype Q males, namely, resulted in 1.3 times increase of the time from phloem from 1st probe. However, comparative analyses of data obtained of males and females feeding on viruliferous plants directly did reveal that males spent 1.4 times longer duration of 1st probe (Table 2, Variable 1) and had 1.4 times more total number of probes before phloem (Table 2, Variable 6) than females. On the other hand, biotype Q males made only 94% of total number of pathway (Table 2, Variable 2) compared with that of biotype Q females.

#### Phloem feeding behaviors

Viruliferous plants exhibited impact on phloem feeding behaviors of both males and females of *B. tabaci* biotype Q. The common effect of viruliferous plants on biotype Q males and females included shortened total duration of 1st salivation (57%, Table 2, Variable 7) and shortened total duration of salivation (41%, Table 2, Variable 9). Viruliferous plants dramatically and specifically reduced total duration of sap ingestion (26%, Table 2, Variable 11) of biotype Q males. In comparison, viruliferous plants reduced greatly the percentage of phloem phase (salivation + ingestion) (37%, Table 2, Variable 14) of biotype Q females. Because of the differential impact of viruliferous plants on biotype Q males and females, females took 2.2 times longer duration of E2 (Table 2, Variable 11) than males. Other phloem variables did not significantly differ between males and females on viruliferous plants.

### Interactions among biotypes, sexes, insect status, and plant status

Table 3 lists the multivariate statistics of comparative analyses on combinational EPG variables to examine possible interactions among various factors. First, EPG variables were combined to examine the effect of one factor in each analysis. For example, the ‘Biotype’ combination compares *B. tabaci* biotypes B and Q, without considering their sexes, insect infestation status and plant infestation status. *B. tabaci* biotypes exhibited major differences in 5 out of 14 variables, including total duration of pathway (Table 3, Variable 3), time to phloem from 1st probe (Table 3, Variable 5), total number of sap ingestion (Table 3, Variable 10), total duration of sap ingestion (Table 3, Variable 11) and total number of salivation after 1st sap ingestion (Table 3, Variable 12). Males and females exhibited differences in 2 out of 14 variables, including the total number of pathway (Table 3, Variable 2) and percentage of phloem phase (salivation + ingestion) (Table 3, Variable 14). Insect infestation status showed significant effect on 3 non-phloem variables [total number of pathway, total number of potential drop (intracellular puncture) and total number of probes before phloem] and 2 phloem variables (total duration of 1st salivation and total duration of salivation) (Table 3, Variables 2, 4, 6, 7 and 9). These changes in feeding behaviors could increase the probability of virus inoculation by viruliferous *B. tabaci* on non-viruliferous plants. Plants infestation status had significant effect in 8 out of 14 variables, including the duration of 1st probe (Table 3, Variable 1), total number of pathway (Table 3, Variable 2), total number of potential drop (intracellular puncture) (Table 3, Variable 4), time to phloem from 1st probe (Table 3, Variable 5), total duration of 1st salivation (Table 3, Variable 7), total duration of salivation (Table 3, Variable 9), total duration of salivation after 1st sap ingestion (Table 3, Variable 13) and percentage of phloem phase (salivation + ingestion) (Table 3, Variable 14).

**Table 3:** Interaction analysis of EPG variables among biotypes, sexes and virus status on *Bemisia tabaci* by multivariate statistics

Variables	*P* value^1^										
	Biotype	Sex	Insect status	Plant status	Biotype * Sex	Biotype * Insect status	Biotype * Plant status	Sex * Insect status	Sex * Plant status	Biotype * Sex * Insect status	Biotype * Sex * Plant status
Non-phloem variables											
1. Duration of 1st probe (min)	0.243	0.106	0.892	**< 0.001**	**0.002**	0.363	0.349	0.490	0.349	0.774	0.799
2. Total number of C (#)	0.538	**0.007**	**0.001**	**0.002**	0.252	0.087	0.381	0.240	0.714	0.491	0.478
3. Total duration of C (min)	**0.029**	0.115	0.399	0.063	0.993	0.307	0.853	0.650	0.520	0.430	0.193
4. Total number of pd. (#)	0.696	0.911	**< 0.001**	**0.007**	0.685	0.052	0.783	0.649	0.926	0.761	0.357
5. Time to 1st E from 1st probe (min)	**0.018**	0.174	0.281	**0.044**	**0.030**	0.484	0.869	0.194	0.312	0.551	0.442
6. Total number of probes before 1st E (#)	0.316	0.407	**0.003**	0.557	**< 0.001**	0.161	0.105	0.264	0.934	0.299	0.397
Phloem variables											
7. Total duration of 1st E1 (min)	0.616	0.491	**0.002**	**< 0.001**	0.951	0.121	0.120	0.417	0.286	0.685	0.959
8. Total number of E1 (#)	0.311	0.474	0.594	0.584	0.710	**0.010**	**0.027**	0.865	0.079	0.586	0.075
9. Total duration of E1 (min)	0.723	0.965	**< 0.001**	**< 0.001**	0.249	0.604	0.365	0.738	0.822	0.108	**0.027**
10.Total number of E2 (#)	**0.043**	0.943	0.098	0.916	0.849	**< 0.001**	**0.018**	0.122	**0.001**	0.820	**0.011**
11. Total duration of E2 (min)	**< 0.001**	0.799	0.469	0.098	0.845	0.929	0.973	**0.043**	0.065	0.087	0.280
12. Total number of E1 after 1st E2 (#)	**0.010**	0.525	0.512	0.690	0.490	**< 0.001**	**0.001**	0.678	**0.026**	0.169	**0.030**
13. Total duration of E1 after 1st E2 (min)	0.138	0.468	0.079	**< 0.001**	0.356	0.990	0.566	**0.007**	**0.046**	0.126	**0.015**
14. Percentage of E (%)^2^	0.173	**0.039**	0.089	**< 0.001**	**0.024**	0.150	0.951	**< 0.001**	**0.047**	**0.002**	0.730

^1^*p* values calculated using multivariate analysis with main effects of biotype (B and Q), sex (male and female), insect status (non-viruliferous and viruliferous), plant status (non-viruliferous and viruliferous) and their interaction. Bolded *P* values are significant at *P*<0.05. ^2^Percentage of E (%) = equals the percentage of total duration of E (E1 + E2). EPG waveforms are as: C = pathway; pd. = potential drop (intracellular puncture); E1 = phloem salivary secretion; E2 = phloem sap ingestion. E = E1 + E2

Second, EPG variables were combined to examine the combinational effect of two factors in each analysis. As shown in Table 3, there was a significant combinational effect of biotypes and sexes on the duration of 1st probe (Table 3, Variable 1), time to 1st phloem from 1st probe (Table 3, Variable 5), total number of probes before phloem (Table 3, Variable 6) and percentage of phloem phase (salivation + ingestion) (Table 3, Variable 14). The combinational effect between biotype and insect infestation status was similar to the combinational effect between biotypes and plant infestation status, both showed a significant effect on total number of salivation (Table 3, Variable 8), total number of sap ingestion (Table 3, Variable 10) and total number of salivation after 1st sap ingestion (Table 3, Variable 12). The combinational effect between sexes and insect infestation status was on total duration of sap ingestion (Table 3, Variable 11), total duration of salivation after 1st sap ingestion (Table 3, Variable 13) and percentage of phloem phase (salivation + ingestion) (Table 3, Variable 14). The combinational effect between sex and plant infestation status was on total number of sap ingestion (Table 3, Variable 10), total number of salivation after 1st sap ingestion (Table 3, Variable 12), total duration of salivation after 1st sap ingestion (Table 3, Variable 13) and percentage of phloem phase (salivation + ingestion) (Table 3, Variable 14).

Combinational effects among three factors were also analyzed. The combinational effects among biotypes, sexes and insect infestation status were on the percentage of phloem phase (salivation + ingestion) (Table 3, Variable 14). The combinational effects among biotypes, sexes and plant infestation status were on total duration of salivation (Table 3, Variable 9), total number of sap ingestion (Table 3, Variable 10), total number of salivation after 1st sap ingestion (Table 3, Variable 12) and total duration of salivation after 1st sap ingestion (Table 3, Variable 13). Among the analyzed factors, CCYV on either insects or plants resulted in remarkable effects on *B. tabaci* feeding behaviors than other factors.

## Discussion

Effects of virus on their vectors can be direct and occur within the vector itself after acquisition. Effects can be also indirect and mediated through infested host plants [51]. CCYV has been reported to be transmitted solely through *B. tabaci* biotypes B and Q [29]. Epidemics caused by CCYV have been rapidly expanding in the field [39, 52]. A recent study from our group revealed that these two biotypes exhibited different ability to spread the virus on cucumber plants [52], and feeding behaviors of two biotypes on cotton plants (non-host plant of CCYV) were directly influenced by the virus [39]. Here we examined the direct and/or indirect impacts of CCYV on the *B. tabaci* feeding behaviors on cucumber plants (host plant of the *B. tabaci* and CCYV).

### Direct effects of CCYV on feeding behaviors of *B. tabaci* biotypes B and Q

In this study, we found that the number of probes and the duration of phloem salivation significantly increased for both *B. tabaci* biotypes after individual vector insects were carried with CCYV. Since the increased number of probes and longer duration of phloem salivation can increase the spread of semipersistently transmitted viruses [53], our data suggest that CCYV can directly increase its vector host ability to spread the virus. This observation is consistent with previous reports that *B. tabaci* with TYLCV were more restless [7] and had more attempted probes and phloem salivation on virus-free plants [5]. Insect vectors of carrying CCYV also increased the frequency of insect feeding-site translocation coupled with larger number of short feeding bouts observed with *B. tabaci* biotype Q, resulting in a greatly increased rate of virus inoculation [13, 54]. Based on our observations that biotype Q was affected by CCYV to a greater degree than biotype B, we speculate that virus infection may result in a greater increase in the ability of biotype Q to spread this virus than biotype B on cucumber plants. This speculation is also supported by a previous report from our group, which has demonstrated that CCYV exerts stronger effects on feeding behaviors of biotype Q than those of biotype B on cotton plants [39]. Shi et al. [55] demonstrated biotype Q transmits *Tomato chlorosis virus* (ToCV), a semipersistently transmitted *crinivirus* virus, in tomato more efficiently than biotype B. Infected vector insects by another virus, TYLCV, also result in biotype Q with stronger ability of spreading virus than biotype B [13]. Currently, biotype Q has become the prevalent strain in the field in most regions of China due to heavy use of insecticides [56]. The replacement of biotype B by biotype Q as the prevalent strain in the field is coincidently with the rapid spread of CCYV in China. Further research is needed to determine whether the increase in the frequency of biotype Q is responsible for the rapid spread of CCYV in the field.

CCYV not only exerts differential impacts on the feeding behaviors of different *B. tabaci* biotypes, but also influences the feeding behaviors of males and females in biotype- and plant-dependent ways. In this study, we found that CCYV directly increases non-phloem probing and phloem salivation more on females than on males of biotype Q, but directly increased phloem salivation more on females than males of biotype B when *B. tabaci* feeds on cucumber plants. Previously, our group has found that CCYV enhances probing and saliva secretion of males more than females of both biotypes B and Q [39]. Ning and collaborators [57] also found TYLCV-infected biotype Q females were more efficient in transmitting virus than corresponding males. The exact factors that influence this complex relationship among CCYV, *B. tabaci*, and plant species and its biological implications remain to be determined. One possible reason for different impacts of CCYV on males and females feeding on different plant species is that host plants for *B. tabaci* and CCYV are not exactly the same. *B. tabaci* can feed on a wide range of plant species whereas CCYV has a narrower plant host range. For example, cotton is a very common host for *B. tabaci*, but is a non-host for CCYV. Differences in feeding behaviors of TYLCCNV-infected *B. tabaci* on TYLCCNV host tobacco plants and its non-host cotton plants have been also reported by other researchers [8]. During the long course of coevolution among insect vectors, virus, and plants, it is not surprising that a complex relationship is formed for best adaptation among the interactive species. In addition, CCYV increased attempt potential drops (pd) (intracellular probing) of biotype Q males and females. The intracellular probing represents stylet puncturing into the plant tissue cells and tasting the cytoplasm often related to the spread of non-persistent viruses [58], while ToCV can be transmitted when *B. tabaci* performed 6 pds (intracellular probing) [53], and intracellular probing (pd waveforms) may also play an important role in CCYV inoculation.

### Indirect effects of CCYV on feeding behaviors of *B. tabaci* biotypes B and Q

The indirect impact of viruliferous cucumber plants on *B. tabaci* feeding behaviors may also enhance viral transmission. Consistent with this possibility, we found that viruliferous cucumber plants could shorten phloem salivation of both *B. tabaci* biotypes, suggesting that viruliferous cucumber plants became more susceptible to *B. tabaci* feeding. Some previous studies have suggested that plant virus can improve the quality of resource for vectors by suppressing the jasmonic acid (JA) defense pathway [59–61]. With weaker plant defense and better nutrients, insect vectors are more likely to be attracted to those viruliferous plants with better performance [12, 62]. *B. tabaci* feeding on TYLCCNV-infected plants have been found with reduced detoxification activity to reduce physiological cost, and can access a more balanced nutrition [63]. In contrast, ToCV-infected tomato reduced nymphal viability and prolonged duration of nymphal stage of biotype B [54]. We also observed differential impacts of CCYV on different *B. tabaci* biotypes indirectly via viruliferous cucumber plants. For example, biotype Q feeding on viruliferous cucumber plants had a shortened phloem sap ingestion. On the other hand, biotype B feeding on viruliferous cucumber plants had prolonged phloem sap ingestion. The shortening of phloem sap ingestion of biotype Q and prolonged duration of phloem sap ingestion of biotype B again suggest that biotype Q may possess greater ability for spreading virus when *B. tabaci* have already been infested with CCYV and biotype B may need more time to acquire virions from viruliferous cucumber plants. This speculation is based on the fact that both viruliferous biotypes B and Q became restless and urgent to transmit the virus out from their bodies. Our observation is consistent with previous reports that biotype Q has stronger ability to spread virus [52].

CCYV also had different impacts on feeding behaviors of males and females of both biotypes B and Q. For example, biotype B males had more non-phloem probing and feeding bouts than biotype B females on viruliferous cucumber plants, suggesting that biotype B males may contribute more to spread virus than those females in the field. On the other hand, biotype Q females were more resilient to the reduction of phloem sieve ingestion caused by viruliferous cucumber plants than biotype Q males. A previous study has reported that aster leafhopper, *Macrosteles quadrilineatus*, females spend more time feeding than males, and are also generally larger than males in order to obtain more nutrients needed to support ovarial development [64]. Pan and collaborators [65] have found that the endosymbiont *Hamiltonella* sp. is higher in TYLCV-infected biotype B and Q females than in males among field populations. Endosymbiotic bacteria may be another factor that affects virus transmission of *B. tabaci* males and females.

In this study, we were unable to examine non-viruliferous *B. tabaci* feeding on viruliferous cucumber plants. The difficulty to carry such an assay is that the time for *B. tabaci* to obtain saturated CCYV is only six hours. Within such a short time the stylet is hardly to have reached the phloem to start feeding. Because of this difficulty, the indirect effector of viruliferous cucumber plants on *B. tabaci* feeding behaviors was estimated using data obtained with viruliferous *B. tabaci* feeding on CCYV-infected cucumber plants. Therefore, the indirect impact of viruliferous plants on *B. tabaci* feeding behavior may have been underestimated in our study.

## Conclusions

In conclusion, we presented the first comprehensive evaluation on CCYV direct and indirect effects on the feeding behaviors of its vector *B. tabaci* to various degrees on biotypes and sexes. The altered feeding behaviors of vectors might be possibly responsible for an increase in the rates of CCYV transmission. Our data revealed that CCYV showed overall stronger direct effects on biotype Q than on biotype B and on females than on males of each biotype. CCYV showed overall stronger indirect effects on biotype B than on biotype Q and on males than on females of each biotype. Our studies gained some new insights towards a better understanding of the interaction among viruses, vectors, and plants; and may lead eventually to improvement of integrated management of *B. tabaci* and the semipersisitenly transmitted plant virus, such as reinforcing plant quarantine, breeding pest-resistant and disease-resistant plant strains, and development of new pesticides.

## Data Availability

All data and materials described in the manuscript are available.

## Abbreviations

C: Pathway waveform
CCYV: Cucurbit chlorotic yellows virus
CLCuV: Cotton leaf curl virus
CMG: Cassava mosaic geminiviruses
E: E1 + E2
E1: Phloem salivary secretion
E2: Phloem sap ingestion
EPG: Electrical penetration graph
JA: Jasmonic acid
*mtCOI*: Mitochondrial cytochrome oxidase I
NVP: Non-viruliferous cucumber plants
NVW: Non-viruliferous *B. tabaci* whitefly
pd.: Potential drop, intracellular puncture
TbCSV: Tobacco curly shoot virus
ToCV: Tomato chlorosis virus
TSWV: Tomato spotted wilt virus
TYLCCNV: Tomato yellow leaf curl China virus
TYLCV: Tomato yellow leaf curl virus
VP: Viruliferous cucumber plants
VW: Viruliferous *B. tabaci* whitefly
